# Human Factors in Airway Management: Designing Systems for Safer, Team-Based Care

**DOI:** 10.3390/jcm14248850

**Published:** 2025-12-14

**Authors:** Manuel Á. Gómez-Ríos, Pavel Michalek, Tomasz Gaszyński, André A. J. Van Zundert

**Affiliations:** 1 Anesthesiology and Perioperative Medicine, University Hospital Complex of A Coruña, 15006 A Coruña, Spain; 2 Clinical Management Section of the Spanish Society of Anesthesiology, Resuscitation, and Pain Therapy (SEDAR), 28003 Madrid, Spain; 3 Science, Research and Education, General University Hospital in Prague, 128 08 Prague, Czech Republic; 4 Department of Anesthesiology and Intensive Therapy, Medical University of Lodz, 90-153 Lodz, Poland; 5 Anaesthesiology, Royal Brisbane and Women’s Hospital, The University of Queensland, Brisbane, QLD 4102, Australia

**Keywords:** human factors, airway management, SEIPS, ergonomics, videolaryngoscopy, patient safety

## Abstract

The increasing complexity of airway management, particularly in high-stakes or emergency settings, demands a holistic approach that accounts not only for technical skill but also for the systems in which clinicians operate. Advances in airway devices such as videolaryngoscopes, videolaryngeal mask airways, flexible intubation scopes, combined techniques, and single-use technologies offer new opportunities for improving outcomes—but also introduce new challenges. This article explores the intersection of human factors and the implementation of new airway devices, using a systems-based lens informed by the SEIPS 3.0 framework. Drawing on recent guidelines, real-world case studies, and design principles, we examine how technological changes affect team dynamics, decision-making, equipment layout, and cognitive load. We also highlight the importance of standardized processes, training, and environmental design in mitigating risk and enhancing performance. Ultimately, we propose actionable strategies to integrate human factors into airway device adoption to improve both patient safety and clinician well-being. This review underscores the fact that embedding human factor principles into the adoption and use of airway technologies is essential to build safer, more resilient, and team-centered airway management systems.

## 1. Introduction

Airway management remains one of the most critical and technically complex responsibilities in clinical practice [1]. Despite advances in training, monitoring, and airway device technology, airway-related complications continue to contribute to preventable morbidity and mortality, particularly in emergency and perioperative settings [2]. The UK’s Fourth National Audit Project (NAP4) [3,4] demonstrated that adverse airway outcomes frequently arise not from a lack of equipment or inadequate technical skill, but from human factors such as communication breakdowns, cognitive overload, role ambiguity, and poor team coordination [5].

A paradigm shift has reshaped the understanding of these issues [6]. Traditional improvement efforts focused on individual technical performance. A contemporary view recognizes airway management as a process embedded within a sociotechnical system in which clinicians, technology, tasks, and organizational structures interact. Human factors science offers practical strategies to optimise this interface [7] by improving situational awareness, reducing cognitive load, supporting shared mental models, and improving ergonomic design [8,9]. These principles extend beyond individual technical skill, emphasizing how teams perform under pressure and how systems can be engineered to make safe performance more likely.

Current literature often addresses technological innovation or training in isolation, with limited integration of the underlying human–system interactions that determine real-world outcomes. This review adopts a systems engineering perspective to synthesize human factors principles with airway device innovation and team performance. It proposes a structured framework to guide the design, implementation, and evaluation of safer airway management systems.

Recent developments—including videolaryngoscopes, video-enabled supraglottic airways [10], combined techniques [11,12], single-use flexible intubation scopes [13], and standardized airway carts—have transformed airway practice [1,14]. Yet the benefits of these technologies depend on how well they are integrated into workflows, how intuitively they can be used during stress, and how effectively they support collaborative decision-making [15]. Embedding human factors principles into device design, procurement, and clinical adoption is therefore essential for achieving consistent safety performance [16,17].

To clearly define the aims and scope of this review, we focus specifically on the intersection between emerging airway technologies and human factors science—an area that, although acknowledged in existing guidelines such as Kelly et al. [16], has not yet been comprehensively synthesized. Rather than replicating guideline recommendations, this review provides a novel systems-based analysis of how new devices reshape cognitive load, team dynamics, and workflow design. Our objective is to delineate how human factors principles can enhance the safe adoption and real-world implementation of modern airway technologies across both routine and high-risk scenarios. Drawing on real-world examples, recent guidelines, and implementation science [1,17,18], it outlines strategies to align people, tools, and environments to achieve consistently safe performance.

## 2. Understanding Human Factors in Airway Management

Applying human factors to airway management requires shifting from a focus on individual technical skill to a systems-based approach [19]. Frameworks such as the SEIPS (Systems Engineering Initiative for Patient Safety) model [20] illustrate how device design, workflow structure, workspace configuration, team communication, and organizational policy collectively shape safety. Integrating these considerations allows teams and institutions to transition from reactive error management to proactive system design that supports resilience, adaptability, and high reliability under pressure [1,21].

### 2.1. Theoretical Framework: Human Factors and the SEIPS Model

Human factors (HF) is the science of optimizing the interactions between people, technology, tasks, environments, and organizations to enhance performance and safety [22]. In airway management, HF aims to reduce cognitive and physical workload, support decision-making under stress, and align workflows with real-world clinical behavior [23,24]. Modern ergonomics extends beyond traditional non-technical skills by incorporating elements such as device usability, workspace layout, equipment standardization, and training environments—factors that strongly influence performance during high-stakes airway events [24,25].

The SEIPS (Systems Engineering Initiative for Patient Safety) 3.0 framework conceptualizes healthcare as a sociotechnical system composed of interdependent components: people, tasks, tools and technologies, physical environments, and organizational structures [26]. Applied to airway management, SEIPS enables systematic evaluation of how these elements interact during routine and emergency scenarios. For example, videolaryngoscope availability shapes task execution, team coordination, and workspace configuration [17], while disorganized airway carts or unfamiliar equipment introduce delays and errors [27].

By integrating HF principles with SEIPS analysis, clinicians and institutions can identify latent system vulnerabilities—such as unclear roles, poorly integrated devices, or suboptimal environment design—and implement proactive strategies that strengthen resilience under pressure [26,28]. This theoretical framework underpins the review’s systems-based perspective on modern airway management.

### 2.2. Lessons from NAP4 and Sentinel Cases

The NAP4 [3,4] remains a landmark investigation into the causes of major airway-related morbidity and mortality. Its findings showed that most severe complications stemmed not from lack of equipment or technical skill, but from failures in planning, communication, teamwork, and timely escalation [29]. These events demonstrate that airway crises are fundamentally systemic, requiring organizational rather than purely technical solutions.

Common patterns identified across incidents included inadequate pre-procedure assessment, failure to anticipate deterioration, delayed transition to rescue strategies, and unclear team roles during crisis management [30]. Cognitive overload, fixation on failing techniques, and inconsistent use of checklists or cognitive aids further compounded decision-making under pressure. On average, more than four human factors failures contributed to each event [29], underscoring how airway harm typically arises from multiple interacting breakdowns rather than a single error.

Sentinel events such as the Elaine Bromiley case [31] highlight the consequences of hierarchical communication, role ambiguity, and the absence of shared situational awareness. Even with experienced clinicians present, lack of coordinated action and delayed strategy change led to catastrophic escalation—illustrating that strong technical skills cannot compensate for weak team structures or communication flow.

#### 2.2.1. FONA (Front of Neck Access) as a Critical Vulnerability Identified by NAP4

NAP4 also exposed major vulnerabilities in the recognition and execution of front of neck access (FONA), particularly during “can’t intubate, can’t ventilate” (CICV) emergencies. Emergency cannula cricothyroidotomy demonstrated high failure rates due to unfamiliar equipment, insufficient training, delayed transition to a surgical airway, and ineffective ventilation. In contrast, surgical cricothyroidotomy was more reliable, emphasizing the need for standardized and practiced techniques.

From a human factors perspective, FONA failures frequently resulted from cognitive overload, fixation on repeated airway attempts, and a lack of shared team mental models regarding when and how to perform a surgical airway. These findings support the importance of clear escalation triggers, standardized FONA kits, accessible equipment layouts, and simulation-based training that incorporates stress exposure and role clarity.

#### 2.2.2. System-Level Implications

Collectively, these lessons reinforce the principle that reliable airway safety arises from systems designed to support human performance [7,32]. Standardized airway planning pathways, structured briefings, crisis cognitive aids, and explicit escalation criteria help teams transition effectively between strategies. Incorporating human factors expertise into guideline development, simulation curricula, and institutional review processes can strengthen the overall resilience of airway management systems.

## 3. Technological Advances in Airway Devices

Recent advances in airway technology offer significant opportunities to improve patient safety, enhance visualisation, and support effective team performance [33,34]. However, the safety benefit of these devices depends not only on their inherent technical sophistication but also on their usability, ergonomic integration, and alignment with cognitive and team-based workflows in real clinical environments [35].

### 3.1. Overview of Emerging Airway Devices

Airway management has evolved rapidly, driven by new technologies aimed at improving safety, efficiency, and team coordination [36,37]. Key innovations include videolaryngoscopes (VLs) [17,38], flexible intubation scopes (FISs) [13], disposable supraglottic airway devices (SGAs) [39], and video laryngeal mask airways (VLMAs) [40,41].

VLs are now widely recommended as first-line devices for both routine and difficult airway management [17]. Their capacity for indirect visualisation improves glottic views and first-pass success [42], while the shared screen supports collaborative problem-solving, supervision, and real-time coaching [43,44,45].

FISs, increasingly available in single-use form, offer rapid deployment, reduced infection risk, and consistent performance across settings, particularly in anticipated or known difficult airways [13].

Video-enabled supraglottic airways, including video laryngeal mask airways (VLMAs) [46], represent an emerging interface between supraglottic ventilation and guided intubation [40]. These devices combine a secure airway seal with continuous glottic visualisation and can facilitate fiberoptic or stylet-guided tracheal intubation through the device. Their use holds promise for both elective and rescue scenarios, though integration into standard practice requires structured training, guideline alignment, and human factors–informed evaluation [47].

Disposable VL blades and SGAs enhance safety and efficiency in high-turnover or resource-limited environments [48].

While technological innovation has been rapid, challenges in ergonomics and usability highlight the need for deliberate evaluation. Device features such as handle geometry, balance, camera orientation, screen placement, and menu interface design can considerably influence task success during high-stress airway interventions. Poorly positioned visual displays may hinder shared situational awareness, while unfamiliar operating modes may contribute to delays or error patterns under cognitive load.

Effective adoption of new airway devices, therefore, requires not only technical proficiency but also systems-based design. This includes standardized equipment layout, harmonized cognitive aids, simulation-based usability testing, and structured team-based training. Aligning device selection and workflow design with human factors principles can support more reliable performance, reduce task complexity, and create clinical environments that enable teams to achieve consistently safe outcomes.

### 3.2. Ergonomic and Usability Considerations

While emerging airway technologies offer considerable clinical advantages, their effectiveness ultimately depends on how well they align with human cognitive and physical performance under real conditions of stress, uncertainty, and time pressure [49]. Usability refers to how intuitively and efficiently a device can be deployed during critical moments, shaped by factors such as interface design, weight distribution, visual display orientation, and tactile feedback [50,51]. The key human factors influencing airway management are summarized in Table 1, which outlines how device design, team coordination, and system factors collectively affect safety and performance.

In airway crises, clinicians rely on rapid perceptual recognition and coordinated motor actions. Device features such as camera orientation, screen position, handle geometry, and instrument guidance channels can either support or hinder performance. Even minor mismatches between expected and actual device behavior can introduce delays, increase cognitive load, and heighten the risk of error—particularly when teams must make split-second decisions under pressure.

For example, videolaryngoscope monitors positioned outside the assistant’s direct line of sight can disrupt shared situational awareness, hindering suction, external laryngeal manipulation, or coordinated introducer placement [45]. Variations in blade curvature, stiffness, or field-of-view depth across devices may require distinct psychomotor patterns, increasing cognitive workload for clinicians working across multiple clinical environments. Likewise, confusing cart layouts or mislabeled connectors can delay airway rescue [52,53].

Human-centered airway system design, therefore, requires more than access to advanced equipment; it demands deliberate configuration of workspace, standardization of equipment layout, and structured training environments that allow teams to develop stable cognitive routines. Simulation-based usability testing should evaluate not only procedural success but also communication efficiency, error recovery, and resilience as complexity escalates.

Consequently, human-centered design and simulation-based usability testing should guide device selection and procurement [16,54,55]. Such approaches evaluate not only technical metrics but also teamwork, error frequency, and response times under pressure [44,56]. Embedding ergonomic and usability principles into device procurement, institutional training programs, and post-implementation evaluation can reduce performance variability and support safer, more predictable airway outcomes. In this way, human factors analysis becomes integral to creating airway systems that are robust, intuitive, and optimized for real-world clinical performance.

### 3.3. Impact on Decision-Making and Team Roles

Advances in airway technology have reshaped both cognitive demands and team dynamics during airway management [57]. Historically, direct laryngoscopy centralized control and decision-making with the intubator, limiting shared visual information and reducing opportunities for collaborative engagement. In contrast, video-enabled devices now allow all team members to observe the airway in real time, supporting shared situational awareness and enabling earlier anticipatory planning, coaching, and corrective action [58,59].

This shared visibility enhances team performance but also introduces new workflow considerations. Clear role definition, verbalization of evolving plans, and proactive communication become essential to prevent task overlapping, hesitation, or diffusion of responsibility. Airway teams must therefore establish structured communication patterns—such as closed-loop dialogue, pre-procedure briefings, and explicit declaration of airway plans and backup strategies—to ensure coordinated performance under pressure.

Device availability and familiarity can influence decision-making pathways. For instance, routine access to videolaryngoscopy may encourage earlier escalation to video-assisted intubation in anticipated difficult airways, while the availability of video laryngeal mask airways or flexible intubation scopes may shape choices in rescue scenarios [1,18,60]. However, increased device choice may also lead to cognitive overload or delayed transition to alternative strategies unless supported by standardized algorithms, cognitive aids, and practice-based training [30,61].

Furthermore, reliance on advanced devices may inadvertently contribute to reduced vigilance or overconfidence, particularly when clinicians assume that video guidance guarantees successful intubation [17,62]. Teams must therefore maintain awareness of evolving conditions, monitor the patient’s physiological stability, and promptly activate rescue pathways when necessary. Human factors’ principles emphasize that safe performance relies on timely decision transitions rather than persistence with a failing strategy.

Optimizing decision-making and team performance requires alignment between equipment selection, role allocation, training, and environmental design. Institutions should ensure standardized training and consistent exposure to core airway devices, incorporating scenario-based and team-based simulation into educational pathways. Structured debriefing and feedback loops should reinforce adaptive behaviors and support safe integration of new devices into practice [16].

To further enhance system reliability, alignment between checklists, equipment layout, and cognitive aids is essential [44]. When teams share a common mental model, use standardized communication strategies, and operate within ergonomically aligned environments, airway management becomes more predictable, resilient, and safe.

## 4. Human-System Interaction in Modern Airway Management: Training, Tasks, and Teamwork

The integration of advanced airway technologies has reshaped how clinicians interact with tools, teams, and tasks. Effective human–system integration requires aligning technical proficiency with communication, coordination, and decision-making processes under pressure [16,61,63].

Video-enabled devices such as videolaryngoscopes and video supraglottic airways have transformed airway management from an individual task to a shared visual and cognitive process [64,65]. This shared situational awareness enhances anticipation and coordination but demands clear team roles and new communication patterns to avoid role ambiguity and cognitive overload [16,53,66,67].

Modern airway emergencies impose substantial cognitive load as clinicians juggle physiology, device setup, task sequencing, and rapid escalation decisions [16,68,69]. While advanced devices can improve visualization and first-pass success, they may also introduce additional demands—such as screen positioning, menu settings, or unfamiliar geometries—that increase workload when training is insufficient [70,71,72,73,74,75,76,77,78,79].

Maintaining situational awareness is essential to managing these demands [5,78,80,81,82,83,84,85,86]. Simulation-based training plays a central role in preparing teams for these cognitive, technical, and behavioral demands [64,87,88,89,90,91]. High-fidelity scenarios allow teams to practice device use, troubleshoot failures, and refine communication strategies, while structured debriefing reinforces crisis resource management and strengthens transfer to real practice. Regular refresher training and local-context simulation ensure that skills remain durable across the entire team [87,92,93,94].

## 5. Designing for Safety: Environment, Equipment, and Workflows

Safety in airway management depends not only on clinician skill or advanced technology but also on the design and alignment of environments, tools, and workflows [7,9,95]. Applying human factors and cognitive ergonomics to the physical setup of spaces, equipment accessibility, and task structure enhances resilience and performance [16]. Design is not merely aesthetic—it is a clinical determinant with measurable effects on outcomes [7,9,63].

### 5.1. Physical Layout of Airway Carts and Clinical Spaces

The physical environment significantly influences performance, especially under stress [29]. Factors such as poor lighting, clutter, noise, and limited workspace contribute to errors and delay in actions, particularly in emergencies requiring rapid coordination [16,86,96].

Airway cart organization offers a practical domain for improvement [16,52]. Traditional “catch-all” trolleys—overstocked and inconsistently arranged—impede efficiency and decision-making [53]. In contrast, carts structured using human factors principles are standardized and task-oriented, with drawers corresponding to airway management steps (e.g., basic airway maneuvers, supraglottic devices, tracheal intubation, front-of-neck access) [97]. Color coding, clear labeling, and intuitive layouts reduce cognitive load and facilitate rapid access.

Design elements such as shared visualization (external VL monitors visible to all), unobstructed pathways, and consistent tool placement have been shown to enhance situational awareness and teamwork [52,64,66]. The ultimate goal is to transform the environment from a passive space into an active enabler of safety [7].

### 5.2. Device Accessibility and Visibility as Decision Prompts

Visibility and proximity of equipment act as powerful cognitive cues that influence decision-making [98]. If a device is out of sight or difficult to access, it is less likely to be used—even when clinically indicated [53,99]. In time-critical airway emergencies, familiarity and reachability drive selection and escalation [100].

Strategic positioning of devices (e.g., bougies, alternative blades, or front-of-neck kits) can both streamline workflow and prompt clinicians to follow algorithmic steps [101]. Organizing airway carts to mirror local protocols or Vortex-based cognitive aids aligns cognitive and physical processes [78]. Likewise, prepackaged “can’t intubate, can’t oxygenate” (CICO) kits promote rapid, structured escalation in crisis scenarios [102,103].

### 5.3. Cognitive Aids and Workflow Standardization

Cognitive aids—checklists, algorithms, and visual flowcharts—are essential safety tools that reduce reliance on memory and foster coordinated team action [1,104]. Their effectiveness, however, depends on integration within the clinical environment and culture.

Successful implementation requires that aids are: (a) Accessible at the point of care (e.g., laminated cards on airway carts or device packaging) [45,104]; (b) Embedded into equipment layout and training, ensuring predictable action sequences [16,78]; and (c) Linked to clearly defined team roles during escalation [66,104].

Designing cognitive aids alongside equipment and workspace configuration is a cornerstone of human-centered systems thinking [9,16]. Institutions that align their carts, checklists, and training under a shared cognitive framework demonstrate improved team coherence and crisis response [52,53,105].

## 6. Case Studies and Implementation Strategies

Implementing human factors in airway management is a dynamic, context-dependent process, not a linear one [106]. By learning from real-world cases, addressing systemic barriers, and leveraging interdisciplinary expertise, healthcare systems can embed safety into their culture and infrastructure [21]. The goal extends beyond preventing failure—to enabling teams to consistently perform at their best, even under pressure [16].

### 6.1. Real-World Examples from Clinical Practice

The integration of human factor principles has yielded measurable gains across diverse settings. Institutions adopting systems-based redesigns report improved teamwork, reduced adverse events, and enhanced clinician satisfaction. At the Royal United Hospitals Bath NHS Trust, NAP4 recommendations prompted a comprehensive redesign of difficult airway carts [107]. Layouts were standardized across departments, drawers color-coded according to Vortex algorithm steps, and external videolaryngoscopy screens installed. Post-implementation audits demonstrated faster intubation times, improved adherence to escalation protocols, and increased team willingness to “speak up” during crises [108].

Similarly, The Children’s Hospital at Westmead (Australia) developed modular, vacuum-sealed airway trolleys organized by patient weight [103]. These enabled rapid access to appropriately sized equipment and minimized cognitive load. The design was incorporated into simulation training, aligning educational and clinical practice environments.

### 6.2. Barriers to Implementation

Despite progress, several obstacles hinder the widespread adoption of human factors–based approaches in airway care [16,53]: (a) Cultural resistance—Task-focused autonomy and skepticism toward standardized tools; (b) Inconsistent training—Variation in staff exposure to simulation and device use; (c) Financial constraints—Procurement decisions prioritizing cost over usability or ergonomics; (d) Siloed operations—Fragmentation across emergency, ICU, anesthesia, and transport teams; (e) Hierarchical structures—Limited cross-departmental governance delaying change; (f) Surgical approval barriers—Resistance to new workflows from procedural specialists.

Overcoming these requires leadership engagement, cross-department coordination, and a shift toward a learning-oriented, psychologically safe culture [21].

### 6.3. Enablers of Effective Integration

Successful implementation depends on several key enablers [16,53,88]: (a) Human Factors Leads—Designated clinicians or ergonomists championing design improvement, training, and system integration; (b) Multidisciplinary Collaboration—Inclusion of nurses, technicians, engineers, and procurement staff to ensure sustainable, user-centered solutions; (c) Simulation-Based Training—Regular, high-fidelity team simulations supporting both technical and non-technical competence; (d) Structured Debriefing and Learning Systems—Routine reflection on adverse and successful cases (Safety-I and Safety-II) promotes continuous adaptation [109]. 

When conducted within a psychologically safe environment, morbidity and mortality meetings can become powerful tools for systems learning and sustained improvement in human factors integration.

## 7. Scalable Models and Frameworks

The hierarchy of controls, widely applied in safety-critical industries, provides a scalable framework for structuring airway safety strategies [110]. This model prioritizes interventions by effectiveness [61]: (a) Designing out hazards (e.g., standardized equipment layout); (b) Building barriers (e.g., checklists, visual cues); (c) Implementing mitigations (e.g., protocols, simulations); and (d) Relying on training and behavior as the final safeguard.

Systems that invert this hierarchy—depending primarily on individual vigilance or exceptional performance—create fragile, error-prone environments [111,112]. In contrast, hospitals that establish system design as the foundation of safety achieve more resilient, consistent, and sustainable airway outcomes [113,114,115].

## 8. Sustainability, Cost, and Global Equity Considerations

Achieving sustainable, cost-effective, and equitable airway management requires systems thinking, not just equipment selection [16,63]. Institutions must balance infection control, environmental impact, usability, and economic feasibility when implementing new technologies [13]. Embedding human factors into procurement and policy—particularly in resource-limited settings—advances safety that is both high-performing and socially responsible [63,114].

### 8.1. Disposable vs. Reusable Technologies

The proliferation of single-use airway devices—such as videolaryngoscope blades, flexible intubation scopes, and supraglottic airways—has improved infection control, a benefit underscored during the COVID-19 pandemic [116,117]. Disposable devices eliminate reprocessing, reduce contamination risk, and ensure rapid availability in high-turnover or remote settings [118]. However, these advantages apply only when single-use devices deliver performance and quality equivalent to their reusable counterparts, which is not consistently the case. For example, some single-use videolaryngoscope blades have demonstrated inferior optical quality and mechanical performance compared with reusable versions. Likewise, their economic and environmental costs remain debated [119,120].

While single-use devices may appear more expensive per unit, life-cycle analyses that include cleaning, maintenance, and storage often narrow the cost gap [121,122]. Additionally, human factors benefits—including faster setup, fewer equipment failures, and simplified training—can offset costs through efficiency and reduced adverse events [123].

Environmental sustainability poses further challenges, as single-use plastics contribute substantial waste and carbon emissions [120,121]. Mitigation strategies include: (a) use of recyclable or biodegradable materials [124]; (b) hybrid models, where high-risk components are disposable but durable parts are reusable [122]; and (c) investment in high-quality reusable devices with ergonomic and safety features comparable to disposables.

Procurement decisions must therefore integrate cost, infection control, usability, and environmental stewardship.

### 8.2. Public Policy Perspective

Professional societies, anesthesiology colleges, and health ministries play a pivotal role in advancing human factors integration [16,125]. Policy priorities should include: (i) national standards requiring ergonomically designed equipment layouts and cognitive aids; (ii) funding for multidisciplinary simulation and usability testing; (iii) embedding human factors criteria in procurement frameworks; and (iv) national campaigns normalizing structured communication and standardized airway carts.

Alignment across emergency, anesthesia, and critical care services ensures interoperable algorithms, shared terminology, and consistent training, making safe performance the system default [1,18].

### 8.3. Procurement Guided by Human Factors

Procurement often emphasizes cost over usability, resulting in technically adequate but impractical devices [126,127]. A human factors–informed procurement process should include: (a) End-user involvement in evaluation and selection [128]; (b) Simulation-based usability testing under realistic stress conditions [129]; and (c) Manufacturer transparency on ergonomic and usability data [126].

Prioritizing devices aligned with team dynamics and workflow reduces procedural variability and enhances safety [50,53].

### 8.4. Implications for Low-Resource Settings

In low- and middle-income countries (LMICs), limited access to equipment and training necessitates context-specific strategies [130,131,132]. Human factors approaches can enhance safety even under constraints [21]. Effective interventions include: (a) low-cost standardized airway carts with ergonomic organization [105]; (b) cognitive aids adapted to local resources [78,104]; (c) affordable, low-fidelity simulation programs targeting communication and escalation skills [88,133]; and (d) reliable maintenance systems extending equipment longevity [131,134].

Here, the focus shifts to designing for resilience—supporting clinicians to succeed despite limitations [21]. Partnerships among local health ministries, NGOs, and global organizations can foster scalable, human factors–driven innovations [135].

### 8.5. The Role of Industry in User-Centered Design

Manufacturers play a crucial role in advancing user-centered design. Many devices still reach clinical use with limited real-world usability testing, resulting in tools that are technically sound but ergonomically flawed [126,128,129,136]. To improve safety, industry must adopt collaborative design, integrating clinicians, human factors experts, and engineers from early development stages [137].

Regulatory authorities—including the U.S. Food and Drug Administration (FDA), the UK Medicines and Healthcare Products Regulatory Agency (MHRA), the European Medicines Agency (EMA) (noting that for medical devices, oversight is delivered through a system of national Notified Bodies rather than a single central authority), and the Australian Therapeutic Goods Administration (TGA)—increasingly mandate usability and human factors evaluations for medical devices [126,138]. However, regulatory compliance alone is insufficient [139]. True innovation demands understanding how devices function within multidisciplinary teams and high-pressure workflows [21].

Moreover, post-market feedback should capture clinician insights on cognitive load, accessibility, and usability—not just adverse events [129,140]. Devices conceived within their operational context are more likely to enable safe, efficient airway management [141].

These policy-oriented recommendations are summarized in Table 2, which outlines key human factors considerations for manufacturers, healthcare systems, and policymakers.

## 9. Future Directions

### 9.1. Research Gaps

Airway management lies at the intersection of technical precision and cognitive complexity [142,143]. While technological advances—such as VLs, VLMAs, and single-use flexible scopes—have improved safety, technology alone is insufficient [143,144]. Sentinel event analyses and implementation research consistently show that outcomes depend equally on system design, team dynamics, and human–machine interaction [19,145].

These emerging tools introduce not only new capabilities but also challenges in workflow, training, and cost [13,74,146,147]. Their effectiveness depends on integration within ergonomic systems supported by cognitive and environmental scaffolding [16,21,148].

Key research and practice priorities include: (a). Embedding human factors expertise in governance—Designate trained human factors leads to align equipment selection, training, and safety processes [16,149,150]: (b) Redefining competence and training—Shift from technical success metrics to team-based scenario performance, reinforced by regular simulation updates [87,88]; (c) Expanding equity-driven innovation– Develop context-adapted airway tools and training for low-resource environments [151,152]; (d) Measuring system-level outcomes—Assess not only intubation success but also time to oxygenation, cognitive load, and situational awareness [70,71,153]; and (e) Learning from success as well as failure—Apply Safety-II principles to identify and replicate resilient performance [154,155].

Equally critical is industry engagement in user-centered innovation [20,144]. Manufacturers must partner with clinicians and human factors experts throughout design, applying usability engineering from prototyping to post-market evaluation. Regulatory standards (e.g., FDA, MHRA) now require such testing, but ethical and clinical imperatives should drive adoption beyond compliance [156]. Devices that are intuitive, ergonomic, and workflow-integrated do more than reduce error—they empower clinicians to perform optimally [143].

Although VL, cognitive aids, and simulation-based training are now established best practices, empirical validation remains uneven. Future studies should move beyond device-focused comparisons toward system-level evaluations that integrate human, technological, and organizational variables. Frameworks like SEIPS 3.0 and the Airway Ecosystem can guide such research toward comprehensive models of performance and resilience.

These knowledge gaps are synthesized in Table 3, which organizes current evidence and future research needs across the SEIPS domains of people, tasks, tools, environment, and organization.

### 9.2. Implementation Roadmap for Human Factors Integration

Embedding human factors into airway management requires a multi-tiered, iterative strategy spanning governance, design, training, and culture. A structured roadmap—anchored in organizational commitment, ergonomic design, interdisciplinary simulation, and feedback—supports the creation of a resilient, continuously learning airway management system. Integration should follow the SEIPS and Airway Ecosystem frameworks, ensuring adaptability across institutions and contexts.

The proposed steps for achieving this integration are summarized in Table 4, which presents a phased roadmap for embedding human factors principles into airway management across governance, design, training, and policy domains.

### 9.3. Artificial Intelligence and National Databases

Future progress depends on harnessing AI and large-scale data systems. A national airway database capturing both clinical and work-system variables—such as team composition, equipment setup, and near misses—would enable comprehensive safety surveillance [157]. Coupled with AI-driven analytics, such platforms could: Identify latent risks and workflow bottlenecks; Predict escalation pathways (e.g., early VL or FIS use for defined phenotypes); Deliver context-specific decision support (e.g., adaptive checklists or Vortex prompts); Detect performance drift and target retraining; and Provide real-time dashboards for system learning [158].

Ensuring privacy, ethical governance, and seamless electronic integration is essential. Combining human factors science with AI enables a shift from retrospective error reporting to proactive system optimization, enhancing safety while reducing cognitive load for clinicians.

## 10. Conclusions

A safe and effective airway is not simply the result of the right tool in the right hands—it is the outcome of the right system, designed with and for the people who use it. Safe airway management extends beyond individual skill or advanced devices—it is the product of well-designed systems, integrated technology, and coordinated teams.

Embedding human factors principles into device selection, workspace design, training, and protocols enhances cognitive performance, situational awareness, and team resilience. Simulation, structured communication, and standardized cognitive aids translate system improvements into real-world safety.

As healthcare embraces increasingly sophisticated technologies, the integration of human factors in science becomes indispensable, not optional. By combining thoughtful design, interdisciplinary collaboration, and systems-based thinking, we can shape a future where airway management is technically precise, predictably safe, team-centered, and globally equitable—a model of care that empowers clinicians and protects patients across all contexts.

## Figures and Tables

**Table 1:** Importance of Human Factors in Airway Management.

Equipment	Human-Centered Systems (HCS)	Airway Incidents/Human Factors Insights
Videolaryngoscope	Ergonomic screen placement; shared visualization enables teamwork; simulation-based usability studies; SEIPS-based workflow integration	NAP4: Miscommunication, role ambiguity, fixation on view; poor screen angle led to delayed intubation in some cases
Video Laryngeal Mask Airway	Combines SGA seal with camera for continuous visualization; design for rescue and training; usability studies ongoing	Early studies: improved rescue success but new failure modes (camera fogging, interpretation errors) under stress
Combined Technique (e.g., VL + rigid or semi-flexible videostylet)	Requires clear coordination and shared mental model; training on hybrid use; ergonomic handling and visibility critical	Case reports: confusion and role overlap during dual-device use; need for cognitive aids to sequence tasks
Flexible Intubation Scope	Design: portable, single-use reduces infection risk; manikin-based simulation for familiarization; emphasis on intuitive controls	NAP4: Delays due to lack of familiarity; errors in scope handling; high cognitive load in emergencies
Adjuncts (e.g., Bougie, SGA, FONA kits)	Cart design and visibility as cognitive cues; color-coded drawers aligned with airway algorithm; integration with checklists	NAP4: Missing or mislaid adjuncts contributed to failed escalation (‘can’t intubate, can’t oxygenate’ delays)
Airway Carts/Environment	Human factors layout—standardized drawers by algorithm steps; clear labeling and visual prompts; accessibility and lighting design	Poorly organized carts delayed response; inconsistent layouts between departments increased error risk
Cognitive Aids & Algorithms (e.g., Vortex, DAS)	Laminated cognitive aids at point of care; embedded into training and workflow; team briefings; closed-loop communication	Failure to follow escalation algorithms; cognitive overload under pressure; lack of shared situational awareness
Training/Simulation	Manikin-based high-fidelity simulation; team-based drills; focus on shared visualization and cognitive load management	Real-world cases show training gaps as root cause of airway incidents; simulation reduces fixation and error rates
Procurement & Design Process	Inclusion of end users; usability and ergonomics validation; feedback loops post-deployment	Devices selected on cost not usability led to underuse or confusion during emergencies

VL = Videolaryngoscope; VLMA = Video Laryngeal Mask Airway; SGA = Supraglottic Airway; FIS = Flexible Intubation Scope; SEIPS = Systems Engineering Initiative for Patient Safety; NAP4 = 4th National Audit Project (UK); FONA = Front-of-Neck Access; DAS = Difficult Airway Society.

**Table 2:** Policy Insight: Designing Airway Devices with Human Factors in Mind. Key policy and procurement strategies to embed human factors principles into the design, evaluation, and adoption of airway management devices.

What the Industry Must Do
Engage human factors specialists and frontline clinicians from the earliest stages of product development. Conduct usability testing in realistic, high-pressure simulation scenarios—not just in lab conditions. Design for the team, not just the individual operator: ensure shared visibility, role compatibility, and ergonomic access. Provide transparent access to usability data and human-system integration reports during procurement evaluations.
What Healthcare Systems Should Demand
Require that airway devices demonstrate human-centered design validation, not just regulatory compliance. Involve end users in procurement and trial phases, with a focus on workflow fit and team compatibility. Establish post-deployment feedback loops to identify design issues, training needs, and latent risks.
The Outcome
Devices that fit the cognitive, procedural, and environmental realities of airway care—reducing error, improving coordination, and enabling safer decisions under pressure. Design is not decoration. It is safety. From the shape of a blade to the layout of a cart, every design choice can support—or sabotage—clinical performance.

**Table 3:** Research Gaps in Airway Management within a SEIPS-Based Framework. Summary of established evidence, persisting uncertainties, and priority areas for research and system redesign.

SEIPS Domain	Known Best Practices/Current Evidence	Areas Requiring Further Validation/Research Gaps
People (Care Team)	Team briefings, role clarity, simulation training, and airway lead programs improve team coordination and situational awareness.	Limited quantitative data linking team training to hard clinical outcomes (e.g., hypoxaemia rates, first-pass success).
Tasks	Use of checklists and standardized algorithms (DAS, Vortex) reduces cognitive load and omissions during airway crisis.	Need for empirical testing of algorithm compliance and decision aids in emergency (non-theatre) settings.
Tools & Technology	Videolaryngoscopy improves glottic view and first-pass success vs. direct laryngoscopy; supraglottic devices and VLMA reduce trauma risk.	Low certainty for major patient outcomes (e.g., hypoxaemia, mortality); insufficient comparative data among VL models and VLMA types.
Physical Environment	Standardized airway trolleys and optimized layout improve equipment retrieval and team ergonomics in simulation.	Lack of multicentre clinical trials linking cart design or layout optimization to patient outcomes; variable transferability across contexts.
Organization/System	Airway governance models and safety culture initiatives improve incident reporting and readiness.	Sparse evidence on system-level ROI, scalability in resource-limited hospitals, and integration with quality improvement frameworks.
Cross-Domain Interactions	Human-factors-based system design (SEIPS, Ecosystem approach) widely endorsed conceptually.	Empirical data missing on how changes in organization or environment modify technology or team performance effects.

SEIPS = Systems Engineering Initiative for Patient Safety; DAS = Difficult Airway Society; VL = Videolaryngoscopy; VLMA = Video Laryngeal Mask Airway. ROI = Return on Investment.

**Table 4:** Implementation Roadmap for Integrating Human Factors into Airway Management. Phased approach for incorporating human factors into airway management through clinical practice redesign, education, simulation, and policy alignment.

Stage/Domain	Key Actions and Strategies	Intended Outcomes
1. Organizational Commitment and Governance	• Establish an Airway Governance Group integrating anesthesia, ICU, and emergency medicine. • Appoint an Airway Lead responsible for system oversight, training, and incident review. • Embed human factors within institutional safety and quality policies.	Institutional accountability; formalized leadership; alignment of human factors with safety metrics.
2. System Design and Standardization	• Develop standardized Airway Carts with uniform drawer layout, color coding, and cognitive aids. • Ensure consistent availability of VL, VLMA, FIS across sites. • Integrate checklists and cognitive tools into workflows. • Perform ergonomic assessments of OR, ICU, and ED layouts.	Improved accessibility and ergonomics; reduced variability; enhanced task efficiency.
3. Human Performance and Team Training	• Implement multidisciplinary simulation focusing on technical and non-technical skills (communication, leadership, situational awareness). • Use structured briefing/debriefing tools for airway events. • Integrate learning from excellence and incident review feedback.	Enhanced team coordination and resilience; improved communication; reduction in preventable errors.
4. Technology Evaluation and Integration	• Evaluate new devices (VL, VLMA, FIS) for usability, setup time, and display ergonomics. • Involve frontline clinicians in procurement decisions. • Establish digital data capture or airway event logs for audit and improvement.	Evidence-based technology adoption; better user-device fit; continuous data-driven learning.
5. Culture and Continuous Learning	• Foster a Just Culture promoting open discussion of successes and near misses. • Conduct regular Airway Safety Rounds and multidisciplinary reviews. • Integrate outcomes into policy updates, simulation scenarios, and procurement decisions.	Sustained safety culture; organizational learning; ongoing refinement of systems.
6. Evaluation and Research	• Define key indicators: equipment readiness, training participation, checklist adherence, patient safety outcomes. • Use Plan-Do-Study-Act (PDSA) cycles for iterative improvement. • Participate in multicenter research assessing system-level interventions.	Objective measurement of progress; scalable, data-supported improvement in airway safety.

VL = Videolaryngoscope; VLMA = Video Laryngeal Mask Airway; FIS = Flexible Intubation Scope; OR = Operating Room; ICU = Intensive Care Unit; ED = Emergency Department; PDSA = Plan–Do–Study–Act.

## Data Availability

The original contributions presented in this study are included in the article. Further inquiries can be directed to the corresponding author.
